# Pancreatic Adenosquamous Carcinoma: A Rare Pathological Subtype of Pancreatic Cancer

**DOI:** 10.3390/jcm11247401

**Published:** 2022-12-14

**Authors:** Qunli Xiong, Zhiwei Zhang, Yongfeng Xu, Qing Zhu

**Affiliations:** Abdominal Oncology Ward, Cancer Center, West China Hospital of Sichuan University, Chengdu 610041, China

**Keywords:** pancreatic adenosquamous carcinoma, histogenesis, genetic features, diagnosis, prognosis

## Abstract

Pancreatic adenosquamous carcinoma (PASC) is a rare pathological subtype of pancreatic cancer (PC), with a worse prognosis than pancreatic ductal adenocarcinoma (PDAC). Due to its rarity, our knowledge of PASC and its biological characteristics are limited. In this review, we provide an overview of the histogenesis, genetic features, diagnosis, treatment, and prognosis of PASC, as well as pancreatic squamous cell carcinoma (PSCC). The information provided here may help to clarify our understanding of PASC and provide useful avenues for further research on this disease.

## 1. Introduction

Pancreatic cancer (PC) is currently the seventh-leading cause of cancer-related death worldwide [1]. In the United States, PC is expected to become the second-greatest cause of cancer-related death by 2030, trailing behind only lung cancer [2]. Patients with PC are typically not diagnosed until they reach advanced stages of the disease, thereby preventing radical surgery options [3]. Pancreatic adenosquamous carcinoma (PASC) is a rare histological subtype of PC, accounting for 0.5–4% of cases [4,5,6,7,8], while pancreatic squamous cell carcinoma (PSCC) is less prevalent, making up approximately 0.5% of cases [7,9]. Since the first case was reported by Herxheimer in 1907 [10], many additional incidences of PASC and PSCC (collectively called PSC) have been reported [11,12,13,14,15,16,17,18,19]. The median overall survival of patients with PSC is often less than a year, with a five-year survival rate of less than 5% [5,7,20]. Currently, because of the low incidence of PSC, very limited research has been published on this condition. Herein, we present a systematic overview of PSC, including its histogenesis, genetic features, diagnosis, treatment, and prognosis, so as to clarify our understanding of the disease and to facilitate the development of effective treatments for this condition.

## 2. Histological Ontogeny

Given that squamous cells are not found in normal pancreatic tissue, the origin of squamous components within pancreatic neoplasms is an important area of research. Two widely accepted hypotheses have guided our current understanding of the origin of squamous components in PSC. The first is the squamous metaplasia theory; the second is the differentiation theory [21,22,23]. The first proposes that squamous metaplasia of the pancreatic ductal epithelium cells occurs due to inflammatory stimulation, followed by subsequent transformation into PSC [23,24,25,26,27]. The causal factors of inflammation can be chronic pancreatitis or obstruction of the pancreatic duct by a clump of tumorigenic cells [28]. Some studies have described an association of PSC with pancreatic intraepithelial neoplasia (PanIN) and proposed a model of adenoma-carcinoma progression. Since cases of pancreatic ductal adenocarcinoma (PDAC) are thought to develop through the adenoma-carcinoma pathway, and, based on the assumption that the squamous component originates from the adenocarcinoma, it is feasible that PSC arises as a developmental sequence, that is, from PanIN to PDAC to PSC [29,30]. Furthermore, during the formation of PASC, keratin K8/18-positive glandular cells have been shown to transdifferentiate into p63-, p40-, and keratin K5/14-positive squamous carcinoma cells [31]. This suggests that the origins of squamous carcinoma arise from metaplastic changes to pre-existing adenocarcinoma. More research is needed to clarify the molecular mechanisms behind the cellular differentiation processes that lead to PSC.

The second theory that is thought to explain the origin of squamous components in PSC is the differentiation theory, which proposes that the same progenitor cell is responsible for the squamous carcinoma and adenocarcinoma components of PASC [32]. Immunohistochemical results have shown that CA19-9, ST-439, and keratin are expressed in both squamous cells and adenoma cells of PSC, providing further evidence in support of this theory [22]. Several recent genetic studies that reported distinct patterns of recurrent copy number aberrations (including 3p21.2–11.1 chromosomal deletions) and common mutations (including to the most frequently mutated genes KRAS and TP53) between the two components of PSC further support the differentiation theory [33,34]. Nevertheless, other theories have been proposed that are less well-adopted, including the collision theory, which states that adenocarcinoma and squamous cell carcinoma represent histologically distinct tumors that arise independently from different sites and eventually combine or fuse [6,22,23,35], as well as the ectopic squamous nest theory [36]. Even in the context of our understanding framed by the squamous metaplasia theory and the differentiation theory, our understanding of the histological ontogeny of PSC remains poorly developed.

## 3. Genetic Features of PASC

In recent years, technological advances have greatly improved our understanding of the pathogenesis of PSC and provided genomic profiles of the disease (Table 1). One study identified mutations at codon 12 in the *KRAS2* gene and loss of the p16 protein in all eight cases of PASC examined [37]. In three cases, loss of the p16 protein was found to be caused by a homozygous deletion in exon 2 of the *P16/CDKN2a* gene. In 2014, Liu et al. screened PASC cases for core nonsense-mediated mRNA decay (NMD) gene mutations and found that most contained somatic acquired mutations in the *UPF1* gene [38]. This represents the first gene known to be selectively mutated in PASC. *UPF1* encodes an RNA helicase that is critical for NMD in a highly conserved RNA degradation pathway. The specific mutation identified in PASC patients alters *UPF1* RNA splicing and interferes with NMD, resulting in upregulated mRNA levels of NMD substrates [38]. This discovery may provide an avenue for diagnosing PASC, and these patients may benefit from treatments targeting NMD.

A whole-exome sequencing (WES) study of 109 pancreatic cancer samples that included 11 PASC cases suggested that the adenosquamous subtype was closely related to an 8q24 amplification (including the *MYC* gene) and a *FLG* gene mutation [39]. In 2017, Xu et al. screened 1033 PC cases and identified two cases of PSCC [40]. To verify the genomic characteristics of PSCC, these authors used high throughput sequencing after in-solution hybrid capture in two PSCC, two PASC, and four PDAC formalin-fixed paraffin-embedded (FFPE) tissues. They identified nine genetic mutations in the PSCC samples, including in the *C7orf70, DNHD1, KPRP, MDM4, MUC6, OR51Q1, PTPRD, TCF4,* and *TET2* genes. A further nine genetic mutations were found in PDAC, including in the *ABCB1, CSF1R, CYP2C18, FBXW7, ITPA, KIAA0748, SOD2, SULT1A2,* and *Z5* genes. Interestingly, the mutations identified in the PASC samples overlapped with those in the PSCC and PDAC tissues. This study may provide valuable information for the pathogenesis of PSCC, as well as for the development of targeted drug therapies.

In 2017, Fang et al. performed whole-exome or whole-genome sequencing (WGS) on 34 PDAC and 17 of PASC and found that the most common single-nucleotide variants in PASC were *P53, KRAS,* and *SMAD4* [33]. Furthermore, a 3p deletion was identified as the most common copy number variant, with the suppressor genes *FHIT, ROBO1, ROBO2*, and *WNT5A* all contained in the 3p chromosome. These findings led to the speculation that the two components of adenosquamous carcinoma might be derived from the same progenitor cell. In 2020, Ma et al. reported that mutations in the *MAP3K1, PDE4DIP*, and *BCR* genes were often found in the germline of PASC patients after performing whole-exon sequencing on 12 pairs of PASC and paracancerous tissues [41]. Germline mutations in *USP6* and somatic mutations in *KRAS, OBSCN*, and *HRNR* were associated with the malignancy of PASC. In order to distinguish the genomic map of PASC and identify therapeutic targets, Lenkiewicz et al. used DNA-content flow cytometry to detect 15 tumor samples contained in five patient-derived xenografts (PDX) [42]. Whole-exome sequencing, genome-wide copy number variation (CNV), and transposable chromatin sequencing (ATAC-seq) were also used to analyze the tumor segments that were purified and classified from these samples. Multiple somatic genomic impairments targeting PASC genome chromatin modulators were found, superposed on characteristic genomic impairments, including *TP53* (87%) and *KRAS* (73%) mutations, *MYC* amplification (47%), and deletion of *CDKN2A* homozygosity (40%). Moreover, ATAC-seq profiles of the three PASC and three PDAC genomes were compared using the flow-sorting PDX model. Chromatin availability genes specific to PASC genomes were identified in all three PASC cases, including the lysine–methyltransferase gene *Smyd2* and the pancreatic cancer stem cell regulatory factor gene *RORC*. In an additional PASC sample, a *FGFR1-ERLIN2* fusion related to focus CNV in two genes was identified. Finally, pan FGFR inhibitors showed significant activity against PASC PDX models derived from a *FGFR1-ERLIN2* fusion. Together, this research suggests that the epigenomic and genomic profiles of PASC provide a novel strategy for targeting this aggressive subtype of PC.

**Table 1:** A comparison of the genetic features between PSC and non-PSC cases.

Reference	Sample Type	Detection Method	Number of Cases		Characteristics	
			Non-PSC	PSC	Non-PSC	PSC
Brody et al. [37]	FFPE tissues	PCR amplification and sequencing;	NA	8	NA	*KRAS2* gene mutations at codon 12 (100%), exon 2 homozygous deletion in the *p16/CDKN2a* gene (37.5%)
Liu et al. [38]	FFPE tissues	Nested PCR and sequencing	29	23	NA	Somatic *UPF1* gene mutations (78.3%)
Witkiewicz et al. [39]	Frozen tissues	WES	98	11	*KRAS, TP53, CDKN2A*, *SMAD4*, *BCLAF1*, *IRF6*, *FLG*, *AXIN1*, *GLI3,* and *PIK3CA* mutations	8q24 locus amplification (including *MYC* gene) and *FLG* gene mutation
Xu et al. [40]	FFPE tissues	In-solution hybrid capture targeting cancer-related genes and high throughput sequencing	2	4	*ABCB1, CSF1R, CYP2C18, FBXW7, ITPA, KIAA0748, SOD2, SULT1A2,* and *ZNF142* mutations	*C7orf70, DNHD1, KPRP, MDM4, MUC6, OR51Q1, PTPRD, TCF4,* and *TET2* mutations
Fang et al. [33]	FFPE tissues	WES, WGS	34	17	*KRAS, TP53* (65%), and *SMAD4* mutations; gains of 3q, 8q (*MYC*), 12p (*KRAS*), and chromosome 19; losses of 3p (*FHIT*), 4p, 6q, 8p, 9p (*CDKN2A*), 17p (*TP53*), and 18q (*SMAD4*)	*KRAS* (100%), *TP53* (88%), and *SMAD4* mutations; gains of 3q, 8q (MYC), 12p (KRAS), and chromosome 19; losses of 3p (*FHIT*) (more frequent in 3p26.3–26.1, 3p25.1–21.31, and 3p21.2–11.1), 4p, 6q, 8p, 9p (*CDKN2A*), 17p (*TP53*), and 18q (*SMAD4*)
Ma et al. [41]	Frozen tissues	WES	NA	12	NA	*MAP3K1* (75%), *PDE4DIP* (58.3%), *BCR* (58.3%), *ALK* (50%), *USP6* (41.7%), *AR* (33.3%), *HLA-A* (33.3%), *SPEN* (33.3%), *KMT2D* (25%), *NUTM2B* (25%), *ZFHX3* (25%), and *MN1* (25%) germline mutations; *TP53* (41.7%), *KRAS* (25%), *HRNR* (25%), and *OBSCN* (25%) somatic mutations
Lenkiewicz et al. [42]	Frozen tissue and FFPE tissues	Whole-genome copy-number variant, WES, and ATAC-seq	NA	15	NA	*TP53* (87%) and *KRAS* (73%) mutations; amplification of *MYC* (47%); homozygous deletion of *CDKN2A* (40%); genes with accessible chromatin including *SMYD2*, *RORC,* and *FGFR1-ERLIN2* fusion

As shown, genomics studies have significantly enhanced our understanding of PSC. The molecular classification and genetic features continue to support clinicians in making accurate, personalized decisions regarding therapeutic options for patients [43]. However, limited genomic profiling has been performed because cases of PSC are rare, compared with PDAC. There is an urgent need for further exploration of the genetic features of PSC.

## 4. Diagnosis

According to World Health Organization guidelines [44], PASC is defined as containing at least 30% squamous carcinoma components combined with adenocarcinoma. However, this classification is contentious because the proportion of squamous carcinoma in PASC is not correlated with clinical characteristics, and evaluation of the percentage is subjective [4,23,27,45]. On this issue, large-cohort studies will be important to clarify the significance of proportional definitions for clinical definitions of squamous carcinoma components in PASC. In addition to its clinical relevance, examination of cellular and pathological features are of importance for the diagnosis of PSC.

### 4.1. Clinical Manifestation and Laboratory Examination

Patients with PSC typically present with abdominal symptoms, obstructive jaundice, and weight loss, which is similar to PDAC [46,47,48,49]. Abnormal function of distant organs and tissues frequently occur following metastasis to other sites, such as the liver, lung [49,50], and even several rare metastatic sites, such as bone and skin [51,52,53]. Laboratory analyses of serum tumor markers have shown that most patients display elevated levels of carbohydrate antigen 19-9 and carcinoembryonic antigen [54]. Humoral hypercalcemia of malignancy may also occur in some patients with PSC, which may be related to elevated levels of serum parathyroid hormone-related protein [26,55,56,57,58].

### 4.2. Imaging Features

Computed tomography (CT) and magnetic resonance imaging of PSC patients usually depict an ill-defined focus in the head of the pancreas, with peripheral ring enhancement and a poorly enhanced central area of extensive central necrosis. Other characteristics of radiography include vessel invasion, tumor thrombus in the portal vein system, peripancreatic tissue invasion, and upstream main pancreatic duct dilatation. These features may provide useful information for diagnosing PSC and predicting disease prognosis [59,60,61,62,63]. A hypermetabolic pancreatic mass with metastatic sites can be detected with positron emission tomography/CT [64,65]. Additionally, PASC tends to show enhanced fludeoxyglucose uptake on both early and delayed phases when compared to PDAC, and the maximum standardized value and mean retention index have the potential to predict malignancy and invasion of PASC [65].

### 4.3. Pathological Features

The typical pathomorphological features of squamous carcinoma often manifest microscopically as definite intercellular bridges and/or focal keratin peal formation in the tumor cells. However, PSC tends to present with poorly differentiated pathologically. Therefore, utilizing immunochemistry to confirm squamous differentiation in the absence of overt keratinization is often necessary. Immunohistochemistry analysis of PSC frequently reveals positive expression of p63 and cytokeratin 5/6, with some PSC cases being positive for nuclear p53 staining and showing loss of the Dpc4 protein [37]. The proliferative capacity of PSC can be determined using Ki-67 staining within the tumor [66]. Moreover, some patients with PSC may present with poorly differentiated sarcomatous components with positive P40 staining histopathologically, and the sarcomatous component may be related to the biological malignancy of PASC [67]. In general, the percentage of grade III and IV tumors is higher in patients with PSC than in those with other pancreatic tumors, and PSC tends to present with a large size, poor differentiation, and node-positivity, indicating that PSC is more aggressive [5,7].

## 5. Prognosis and Treatment

Compared to PDAC, patients with PSC tend to suffer a worse survival rate (Table 2). For example, Imaoka et al. reported that the median overall survival of patients with PDAC was 15.7 months, while that of patients with PSC was 8.3.(*p* = 0.026) [68]. In the study cohort of Boyd et al., the survival of PSC patients tended to be worse than that of PDAC patients even though there was no statistic difference (*p* = 0.08); there was also no different in the one-year survival rate [5]. Due to the poor prognosis of PSC, finding effective treatments is a top priority [69]. Currently, the treatment of PASC is similar to that of PDAC. Radical surgery remains the only way to cure a tumor, and the same is true for PSC patients in which surgery is the strongest predictor of survival. The median survival and year-survival rate of patients with PSC undergoing surgery, especially those with R0 surgical resection, are improved compared to patients without radical resection [5,70,71,72]. For example, the postoperative median survival of patients with PSC who underwent radical resection could be as high as 12 months [5,73]. However, the prognosis of patients with pancreatectomy for PSC can still be poor [35,74,75,76]. Okabayashi et al. reported a survival of only 6.8 months after surgical resection of PSC [75]. Aside from surgery, patients with PSC can also benefit from other treatment methods, especially chemotherapies [43,73,77,78,79]. A study of 515 cases of PSCC in which 48% of the cohort who received either chemotherapy, radiation therapy, or both, found that PSC patients in stage IV treated with chemotherapy had a better overall survival than those without chemotherapy, while adjuvant chemotherapy did not improve overall survival in localized early stages [80]. Statistical analyses from 38 patients with PASC demonstrated that the improved survival of PASC patients with a tumor size of at least 3 cm and vascular or perineural invasion was related to adjuvant chemoradiation [4]. Furthermore, retrospective analyses of PASC patients suggested that PASC might be responsive to combined treatments, especially with the inclusion of platinum agents [81,82]. A recent case report documented the success of capecitabine therapy lasting 34.6 months in a 51-year-old female with PASC, who had received surgical resection after experiencing tumor recurrence during initial treatment with gemcitabine [83]. This case highlights that early recurrence during adjuvant chemotherapy for PASC may be compatible with a subsequent exceptional response to second-line chemotherapy. Additional studies have shown that neoadjuvant chemotherapies may improve the successful rate of pancreatectomy surgeries, thereby boosting the prognosis of patients with PSC [84,85,86]. Finally, immunotherapy has become a promising treatment option, with immune checkpoint inhibitors providing therapeutic potential when selectively targeting the squamous components of PASC that express PD-L1 [87].

The low incidence of PSC and the lack of understanding around its biological features prohibit the possibility of clinical trials being carried out in patients with PSC. Therefore, current treatment strategies for PSC are based on clinical studies of PDAC. Although surgery, chemotherapy, and other therapies have improved the prognosis of PSC and long-term survival of patients is now possible [89,90], the effectiveness of these treatments needs to be improved. A comprehensive treatment paradigm that includes surgery, chemotherapy, radiotherapy, and other methods is needed to significantly improve the prognosis of patients with PSC [91,92,93,94,95,96,97].

## 6. Conclusions

Rare subtypes of pancreatic cancer present with unique clinicopathologic characteristics and display a variety of tumor-specific biological features [98,99,100]. The low incidence of PSC has been limiting for our ability to study this disease. In order to gain a more comprehensive understanding, larger-cohort clinical studies and more fundamental research on the mechanisms underlying the genesis and development of PSC are needed. We propose four potential directions for future PSC research. Firstly, we recommend the collection and analysis of large cohorts of adenosquamous carcinomas with long-term survival and in which tumor site is not limited to the pancreas, in order to identify the similarities between these cases. Secondly, the utilization of advanced technologies, such as single-cell sequencing and spatial transcriptomics, will be critical to uncover more genetic features and the histogenesis of PSC and may provide new therapeutic targets. Thirdly, targeted drugs may be developed based on the unique molecular characteristics of PSC and potential targets. Finally, the establishment of systematic databases and standard therapeutic strategies for rare histology subtypes of pancreatic neoplasms (not only limited to PSC) will be essential to aid clinical decision-making and help to provide more accurate prognostic information for patients with PSC.

## Figures and Tables

**Table 2:** The comparison of outcomes between PSC and PDAC from published studies.

References	Year of Study	Number of Patients		Median Survival (Months)			Postoperative Median Survival (Months)			One-Year Survival Rate (%)	
		PDAC	PSC	PDAC	PSC	*p* Value	PDAC	PSC	*p* Value	PDAC	PSC
Boyd et al. [5]	1988–2007	45,693	415	5	4	0.08	16	12	<0.0001	24.7	21.2
Imaoka et al. [68]	2001–2011	56	28	15.7	8.3	0.026	NA	NA	NA	63.5	38.7
Simone et al. [6]	2001–2011	230	7	20.4	8.2	0.23	NA	NA	NA	0 (three-year survival rate)	19 (three-year survival rate)
Katz et al. [73]	2000–2007	14,746	95	Not mentioned	4	0.41	NA	12	NA	NA	NA
Voong et al. [4,88]	1993–2005; 1986–2007	908	38	NA	NA	NA	17.9	10.9	NA	NA	NA

## Data Availability

Not applicable.
